# The Hepatoprotection by Oleanolic Acid Preconditioning: Focusing on PPAR*α* Activation

**DOI:** 10.1155/2018/3180396

**Published:** 2018-04-02

**Authors:** Wenwen Wang, Kan Chen, Yujing Xia, Wenhui Mo, Fan Wang, Weiqi Dai, Peiqin Niu

**Affiliations:** ^1^Department of Gastroenterology, Shanghai Tenth People's Hospital, Tongji University School of Medicine, Shanghai 200072, China; ^2^Department of Gastroenterology, Minhang Hospital, Fudan University, Shanghai 201100, China; ^3^Department of Oncology, Shanghai General Hospital, Shanghai Jiaotong University School of Medicine, Shanghai 200080, China; ^4^Department of Gastroenterology, Zhongshan Hospital, Fudan University, Shanghai 200032, China; ^5^Shanghai Institute of Liver Diseases, Zhongshan Hospital, Fudan University, Shanghai 200032, China; ^6^Shanghai Tenth People's Hospital Chongming Branch, Tongji University School of Medicine, Shanghai 202157, China

## Abstract

**Objective:**

Previous studies have characterized the hepatoprotective and anti-inflammatory properties of oleanolic acid (OA). This study aimed to investigate the molecular mechanisms of OA hepatoprotection in concanavalin A- (ConA-) induced acute liver injury.

**Materials and Methods:**

ConA (20 mg/kg) was intravenously injected to induce acute liver injury in Balb/C mice. OA pretreatment (20, 40, and 80 mg/kg) was administered subcutaneously once daily for 3 consecutive days prior to treatment with ConA; 2, 8, and 24 h after ConA injection, the levels of serum liver enzymes and the histopathology of major factors and inflammatory cytokines were determined.

**Results:**

OA reduced the release of serum liver enzymes and inflammatory factors and prevented ConA mediated damage to the liver. OA elevated the expression levels of peroxisome proliferator-activated receptor alpha (PPAR*α*) and decreased the phosphorylation of c-Jun NH2-terminal kinase (JNK).

**Conclusion:**

OA exhibits anti-inflammatory properties during ConA-induced acute liver injury by attenuating apoptosis and autophagy through activation of PPAR*α* and downregulation of JNK signaling.

## 1. Introduction

The liver is a metabolic and immunological organ that performs a variety of functions, including deoxidation, glycogen storage, and secretory protein synthesis. The onset of liver injury may occur in response to various factors like excessive alcohol use, infections, chemical drugs, and autoimmune disorders [1]. Concanavalin A (ConA) is a mitogenic plant lectin extracted from* Canavalia brasiliensis* [2] that has been used to model liver injury* in vivo*. ConA-induced liver injury is a well-established model to explore the pathogenesis of liver injury [3–5], as it is characterized by elevated liver enzymes and inflammatory cytokines, such as TNF-*α*, IL-1*β*, IL-4, and IL-6, leading to the activation of T cells, sinusoidal endothelial cells (SECs), and Kupffer cells (KCs) [3, 6–9]. Furthermore, animal studies have shown that apoptosis and autophagy are associated with ConA-induced liver injury in a c-Jun NH2-terminal kinase- (JNK-) dependent manner [10–13]. Oleanonic acid (3*β*-hydroxyolean-12-en-28-oic acid, OA) is a pentacyclic terpenoid found in the form of free acids and triterpenoid saponin glycosides in plants [14, 15]. Previous studies have demonstrated that OA and its derivatives possess anticancer, antidiabetic, anti-HIV, antioxidant, and anti-inflammatory [16–20] properties. However, OA's most well characterized properties are hepatoprotective, and it alleviates acute chemical-induced liver injury and chronic liver fibrosis and cirrhosis [21, 22]. OA has even been used as an over-the-counter drug to treat liver disorders in China [15]. Though the exact mechanistic targets modulated by OA during liver injury are unknown, several groups have suggested that ERK, JNK, PI3K/Akt, and Nrf2 may be involved [23–27].

Peroxisome proliferator-activated receptors (PPARs) are a group of nuclear receptors which function as transcription factors and regulate gene expression by binding their heterodimeric partner retinoid X receptors at specific PPAR-response elements [28]. PPARs used to be predominantly associated with lipid metabolism, but follow-up studies have suggested that PPARs participate in the regulation of inflammation and immunity [29, 30]. PPAR*α*, abundantly expressed in hepatocytes, the heart, muscle tissues, adipose tissues, and the kidney, is one of three identified isoforms of PPARs (*α*, *β*/*δ*, and *γ*). Studies have confirmed that PPAR*α* is a transcriptional activator of lipid metabolism and also a suppressor of acute phase immunity in both humans and rodents [31]. PPAR*α* is also associated with alterations in the development of both B and T lymphocytes [30, 32].

This study aimed to investigate the role of OA in ConA-induced liver injury and the underlying signaling pathways associated with its hepatoprotective properties. Based on the mechanisms of hepatoprotection of OA during liver injury induced by other small molecules like phalloidin [33], CCl4 [34], and acetaminophen [25], we hypothesized that OA attenuated ConA-induced liver injury in a PPAR*α*- and JNK-dependent manner.

## 2. Materials and Methods

### 2.1. Chemicals and Reagents

ConA and OA were obtained from Sigma-Aldrich (St. Louis, MO, USA). The alanine aminotransferase (ALT) and the aspartate aminotransferase (AST) microplate test kits were obtained from the Nanjing Jiancheng Bioengineering Institute (Jiancheng Biotech, China). The enzyme-linked immunosorbent assay (ELISA) kits were acquired from eBioscience (San Diego, CA, USA). The RNA polymerase chain reaction (PCR) kit was purchased from Takara Biotechnology (Dalian, China). The antibodies for PPAR*α*, JNK, p-JNK, TRAF2, Bax, Bcl-2, LC3, Beclin 1, and caspase-3 were provided by Proteintech (Chicago, IL, USA). The IL-1*β*, IL-6, and TNF-*α* antibodies were from Abcam (Cambridge, MA, USA). The TdT-mediated dUTP nick end labeling (TUNEL) apoptosis assay kit was from Roche (Roche Ltd, Basel, Switzerland).

### 2.2. Animals

Male Balb/c mice, 6–8 weeks old, weighing 23 ± 2 g, were supplied by Shanghai SLAC Laboratory Animal Co. Ltd. (Shanghai, China). The mice were housed in plastic cages at a temperature of 24°C with a 12 h light-dark cycle and were provided with food and water ad libitum. All our animal experiments conformed to the National Institutes of Health Guidelines and were approved by the Animal Care and Use Committee of Shanghai Tongji University. No animals died or became severely ill prior to reaching our experimental endpoints.

### 2.3. Experimental Design

OA was prepared as an injectable suspension with olive oil and was administered once daily subcutaneously for 3 consecutive days with doses of either 20, 40, or 80 mg/kg prior to ConA injection. ConA was dissolved in saline to a concentration of 2.5 mg/mL and injected at a dose of 20 mg/kg [35, 36] in the caudal vein to induce acute liver injury.

The mice were randomly divided into seven groups:

1. Normal control group (*n* = 6): mice were given saline.
2. Oil control group (*n* = 6): mice were given an equal volume of olive oil.
3. OA group (*n* = 6): mice were given 40 mg/kg OA suspension.
4. ConA group (*n* = 18): mice were injected with 20 mg/kg ConA via caudal vein.
5. ConA + OA 20 mg/kg group (*n* = 18): mice were given 20 mg/kg OA for 3 days before ConA injection.
6. ConA + OA 40 mg/kg group (*n* = 18): mice were given 40 mg/kg OA for 3 days before ConA injection.
7. ConA + OA 80 mg/kg group (*n* = 18): mice were given 80 mg/kg OA for 3 days before ConA injection.

### 2.4. Biochemical Analysis

Blood samples were collected by retroorbital bleeding, and the collected blood was centrifuged at 3000 r/min for 10 min at 4°C to obtain sera. The activities of serum alanine aminotransferase (ALT) and aspartate aminotransferase (AST) were determined using commercial assay kits. The levels of IL-1*β*, IL-6, and TNF-*α* were determined using ELISA kits, per manufacturer's instructions.

### 2.5. Histopathology and Quantification of Liver Injury

Liver tissues were removed from a portion of the left lobe, fixed in 4% paraformaldehyde, embedded in paraffin, sliced to 5-micron thick sections, and stained with hematoxylin and eosin (H&E). Inflammation and tissue damage were assessed using a light microscope. 5 fields (200x magnification) were evaluated from 4 to 6 individual animals per group by an experienced pathologist. Liver sections were blind to observer. The percentages of necrotic area were used for statistical analysis [37]. Based on the severity and distribution of the necrosis, the overall grade of necrotic lesion was evaluated by a scoring system from 0 to 4: 0: none; 1: mild; 2: moderate; 3: marked; 4: severe to diffuse.

### 2.6. Immunohistochemistry

Liver tissues were prepared as paraffin-embedded sections, dewaxed in xylene, and dehydrated in ethanol. Antigen retrieval was achieved using citrate buffer and incubation at 95°C water for 20 min. To block the activity of endogenous peroxidases, the sections were incubated with 3% hydrogen peroxide for 10 min at 37°C. Nonspecific binding was blocked with 5% bovine serum albumin for 30 min. Liver sections were then incubated overnight at 4°C with the following primary antibodies and dilutions: IL-1*β* (1 : 100), IL-6 (1 : 100), TNF-*α* (1 : 100), PPAR*α* (1 : 50), phospho-JNK (1 : 100), LC3 (1 : 50), Beclin 1 (1 : 50), Bcl-2 (1 : 100), and Bax (1 : 100). The slices were then washed three times and incubated with secondary antibodies. A diaminobenzidine kit was used to measure antibody binding under a light microscope. The ratios of stained and total area were calculated using Image-Pro Plus software (version 6.0).

### 2.7. TUNEL Staining

The prepared paraffin sections were dewaxed in xylene for 5–10 min twice and dehydrated with ethanol. Proteinase K without DNase was added at a concentration of 20 micrograms/mL for 15–30 min. TUNEL reaction buffer was added to the slices after washing according to the manufacturer's protocols, and the sections were observed under a light microscope to determine the number of apoptotic cells.

### 2.8. Quantification of mRNA by Reverse Transcription PCR (RT-PCR)

Total RNA in liver tissues was reverse-transcribed into cDNA using the reverse transcription kit (TaKaRa Biotechnology, China) and the resulting cDNA was used for real-time PCR analysis using SYBR Premix EX Taq (TaKaRa Biotechnology, China) with a 7900HT fast real-time PCR system (Applied Biosystems, CA, USA). Oligonucleotide primer sequences are listed in Table 1. The relative expression levels were calculated using the 2^−ΔΔCt^ method and normalized to *β*-actin.

### 2.9. Western Blot Analysis

Total protein was extracted using radio immunoprecipitation assay lysis buffer with protease inhibitors (PI) with phenylmethane-sulfonyl fluoride from liver tissues and stored at −80°C. Protein concentrations were determined using a BCA protein assay kit according to the manufacturer's instructions (Kaiji, China). Equivalent amounts of total protein (120 microgram) were separated in 7.5%–12.5% SDS-polyacrylamide gels and then transferred to polyvinylidene fluoride membranes. Nonspecific binding was blocked with phosphate-buffered saline (PBS) containing 0.1% Tween 20 (PBST) and 5% nonfat milk (dissolved with PBS) for 1 h, and the membranes were incubated overnight at 4°C with the following primary antibodies and dilutions: *β*-actin (1 : 1000) LC3 (1 : 1000), Beclin 1 (1 : 500), Bcl-2 (1 : 1000), Bax (1 : 500), caspase-3 (1 : 500), JNK (1 : 1000), phospho-JNK (1 : 500), and TRAF2 (1 : 1000). Membranes were washed with PBST three times and incubated with horseradish peroxidase-conjugated anti-rabbit or anti-mouse secondary antibodies (1 : 2000) for 1 h at room temperature. Finally, membranes were washed three times and scanned with the Odyssey two-color infrared laser imaging system.

### 2.10. Statistical Analysis

All data are expressed as means ± standard error. The differences between groups were analyzed using one-way analysis of variance. A *P* value < 0.05 was considered as statistically significant. Statistical analyses were performed using Graphpad Prism Software (version 6.0, San Diego, CA, USA).

## 3. Results

### 3.1. Oleanolic Acid (OA) Is Safe and Tolerable in Mice

**Figure 1:**
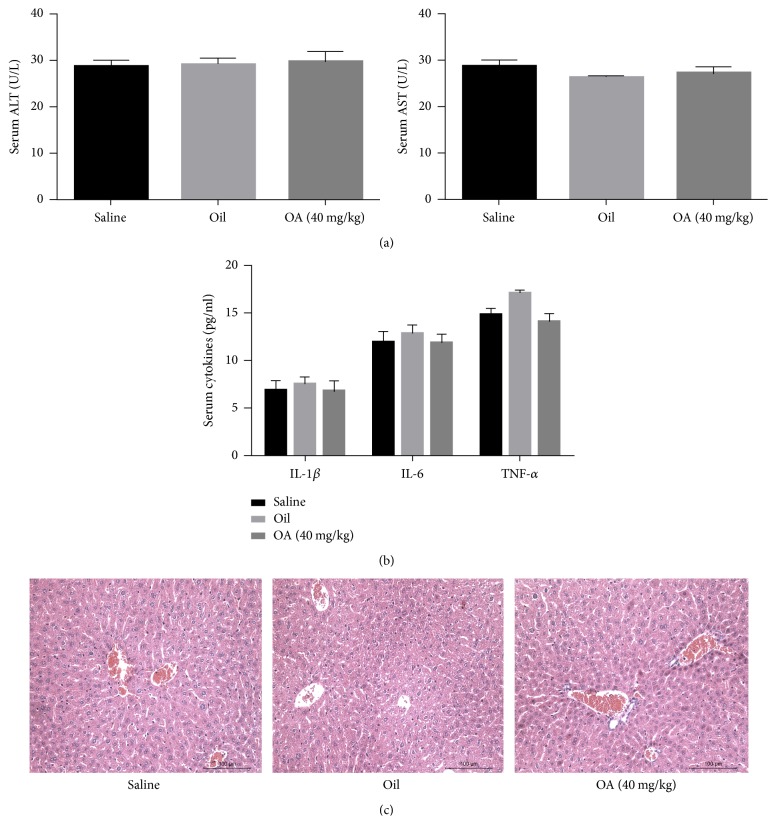
*OA is safe and tolerable in mice*.* Notes*. (a, b) The levels of serum ALT and AST (a) and of IL-1*β*, IL-6, and TNF-*α* (b) are expressed as means ± standard error (*n* = 6, *P* > 0.05). (c) Representative hematoxylin and eosin (H&E) stained sections of livers from saline, olive oil, and OA treated mice (200x magnification).

To determine the safety and tolerability of OA, 18 mice were given equal volumes of either saline, olive oil, or OA (40 mg/kg) suspension for 3 days and then sacrificed to examine signs of liver dysfunction. There were no significant differences in the expression levels of liver enzymes and inflammatory cytokines between the three groups (Figures 1(a) and 1(b)). Liver biopsies showed no pathological and morphological changes in response to OA (Figure 1(c)). Thus, OA appears to be safe and tolerable in these mice.

### 3.2. OA Alleviates Liver Injury Induced by ConA

**Figure 2:**
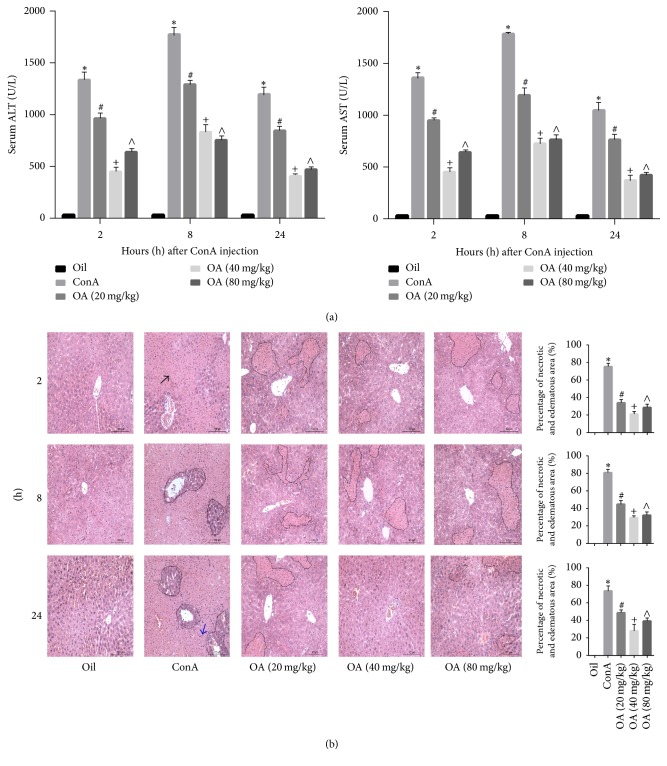
*Pathological liver injury induced by ConA is attenuated by OA*.* Notes*. (a) Serum aminotransferase activities were determined at 2, 8, and 24 h after intravenous injection of 20 mg/kg ConA. Olive oil injected animals were used as a control. (b) Representative hematoxylin and eosin (H&E) staining of livers. Scale bar: 200 microns. Necrotic area was outlined with dotted line, black arrows indicate representative areas of injury, and blue arrows indicate leukocyte adhesion to vascular endothelium. The percentage of necrotic and edematous areas on the basis of H&E liver sections was analyzed with Image-Pro Plus 6.0 (original magnification, ×200). Data were presented as means ± standard error (*n* = 6; ^*∗*^*P* < 0.05 for oil versus ConA; ^#^*P* < 0.05 for ConA + OA (20 mg/kg) versus ConA; ^+^*P* < 0.05 for ConA + OA (40 mg/kg) versus ConA; ^∧^*P* < 0.05 for ConA + OA (80 mg/kg) versus ConA).

We found that, after ConA injection, SECs, KCs, and CD4^+^ Th cells were activated, resulting in elevated levels of cytokines and edema and necrosis in hepatocytes [6, 7]. The levels of ALT and AST were also increased in response to ConA (Figure 2(a)). The most significant increases occurred at 8 h, and OA markedly alleviated the activities of these transaminases. The reduction in ConA-dependent changes was most prominent in the intermediately dosed mice (40 mg/kg). As shown in Figure 2(b), furthermore, liver tissue in oil group showed well preserved hepatic architecture with intact liver lobules. 8 hours after ConA administration, ConA group showed diffuse necrosis, congestion, and partially severe inflammation, and the integrality of hepatic lobules was destroyed. In OA pretreatment group, narrowed necrotic area, slight congestion, and milder lymphocytic accumulation were seen compared to treatment with ConA alone. The patterns of liver tissue of these five groups in 2 h and 24 h were similar to the 2 h. Again, the intermediately dosed mice (40 mg/kg) showed the least amount of necrosis.  Table 2 showed the pathological score of liver injury 8 h after ConA administration. These results suggest that pretreatment with OA can attenuate ConA-induced liver injury in mice.

### 3.3. OA Pretreatment Reduces the Production of TNF-*α*, IL-1*β*, and IL-6 in ConA-Induced Liver Injury

**Figure 3:**
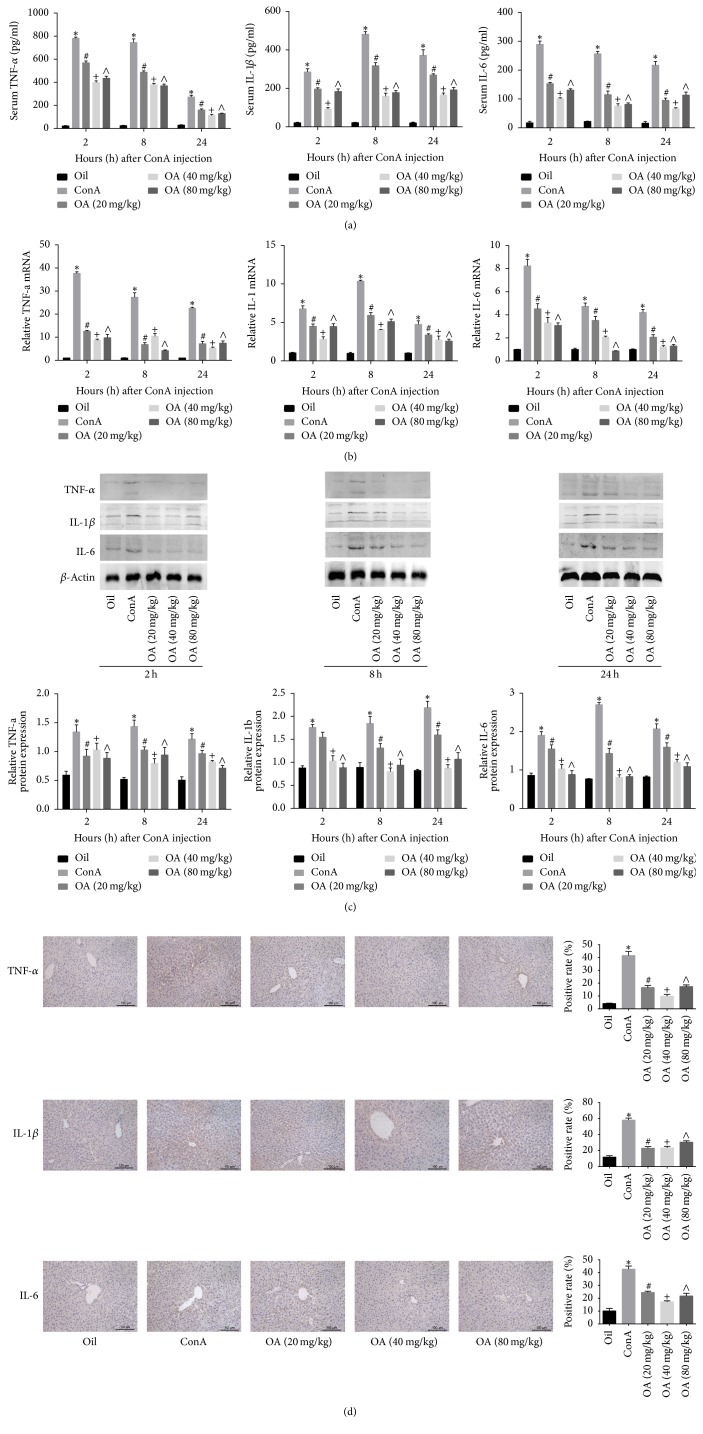
*OA attenuates the production of inflammatory cytokines in ConA-induced liver injury*.* Notes*. (a, b) The relative plasma (a) and mRNA (b) TNF-*α*, IL-1*β*, and IL-6 levels at 2, 8, and 24 h after ConA injection and pretreatment with low (20 mg/kg), intermediate (40 mg/kg), and high (80 mg/kg) doses of OA. (c) Protein levels of TNF-*α*, IL-1*β*, and IL-6 were evaluated by western blotting and the gray values were calculated. (d) Immunohistochemistry staining (200x magnification) of TNF-*α*, IL-1*β*, and IL-6 in liver tissues after 8 h of treatment with ConA. The ratio of brown area to total area was analyzed with Image-Pro Plus 6.0 (*n* = 6; ^*∗*^*P* < 0.05 for oil versus ConA; ^#^*P* < 0.05 for ConA + OA (20 mg/kg) versus ConA; ^+^*P* < 0.05 for ConA + OA (40 mg/kg) versus ConA; ^∧^*P* < 0.05 for ConA + OA (80 mg/kg) versus ConA).

Expectedly, the expression levels of inflammatory cytokines were all elevated after ConA injection. The mRNA and protein expression levels of TNF-*α* and IL-6 peaked at 2 h and then declined at 8 and 24 h, while the levels of IL-1*β* peaked at 8 h. Pretreatment with OA markedly decreased cytokines expression levels compared to ConA treated mice, and the medium dose (40 mg/kg) had the most potent effect on attenuating inflammation (Figures 3(a) and 3(b)). Furthermore, western blotting and TNF-*α*, IL-1*β*, and IL-6 immunohistochemistry analysis also confirmed that OA significantly reduced the production of TNF-*α*, IL-1*β*, and IL-6 on protein level (Figures 3(c) and 3(d)).

### 3.4. OA Reduces Autophagy and Apoptosis in ConA-Induced Liver Injury

**Figure 4:**
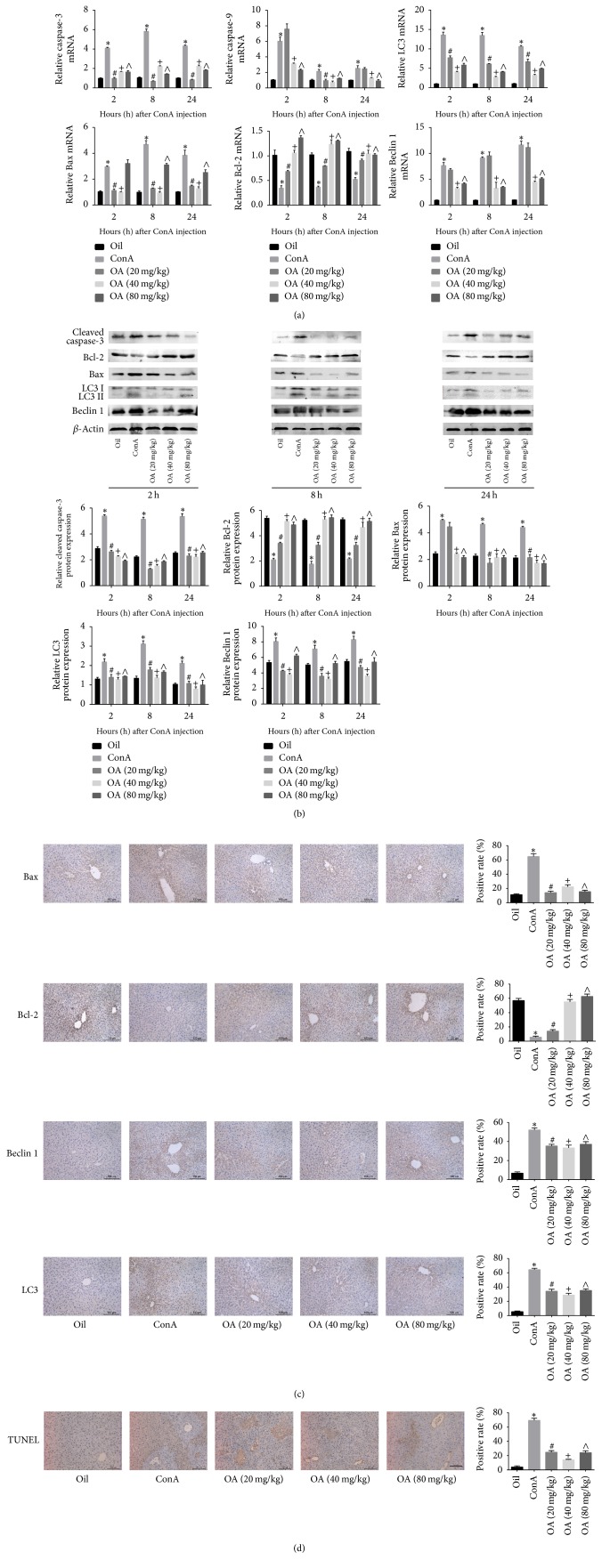
*OA regulates autophagy and apoptosis in ConA-induced liver injury*.* Notes*. (a) mRNA levels of caspase-3, caspase-9, Bcl-2, Bax, LC3, and Beclin 1 were determined using real-time PCR. (b) Protein expression of caspase-3, Bcl-2, Bax, Beclin 1, and LC3 was evaluated by western blot. (c) Representative immunohistochemistry images (200x magnification) show the expression levels of Bax, Bcl-2, Beclin 1, and LC3 at 8 h of exposure to ConA. (d) TUNEL staining (200x magnification) of liver tissue at 8 h represents apoptotic cells (*n* = 6; ^*∗*^*P* < 0.05 for oil versus ConA; ^#^*P* < 0.05 for ConA + OA (20 mg/kg) versus ConA; ^+^*P* < 0.05 for ConA + OA (40 mg/kg) versus ConA; ^∧^*P* < 0.05 for ConA + OA (80 mg/kg) versus ConA).

We assessed the activation of apoptosis and autophagy by measuring the expression levels of caspase-3, caspase-9, Bcl-2, Bax, Beclin 1, and LC3. Previous studies have identified that caspase-3, caspase-9, Bcl-2, and Bax played important roles in regulating apoptosis, and Beclin 1 and LC3 are known mediators of autophagy. The expression levels of Bcl-2, an antiapoptotic marker, were downregulated in response to ConA, and OA pretreatment abrogated this effect. Bax, caspase-3, caspase-9, Beclin 1, and LC3 were all highly expressed in mice given ConA, and these levels were reduced in response to OA pretreatment (Figures 4(a) and 4(b)). Immunohistochemistry analysis and TUNEL staining confirmed these results (Figures 4(c) and 4(d)). These findings suggest that OA regulates autophagy and apoptosis and protects hepatocytes from pathological damage induced by ConA.

### 3.5. OA Inhibits ConA-Induced Liver Injury via Activation of PPAR*α* and Suppression of JNK Signaling

Previous studies have suggested that attenuation of JNK signaling could effectively alleviate ConA-induced liver injury and that PPAR*α* participates in the regulation of inflammation and immunity. Therefore, we hypothesized that PPAR*α* and JNK signaling are involved in the hepatoprotective effects of OA.

**Figure 5:**
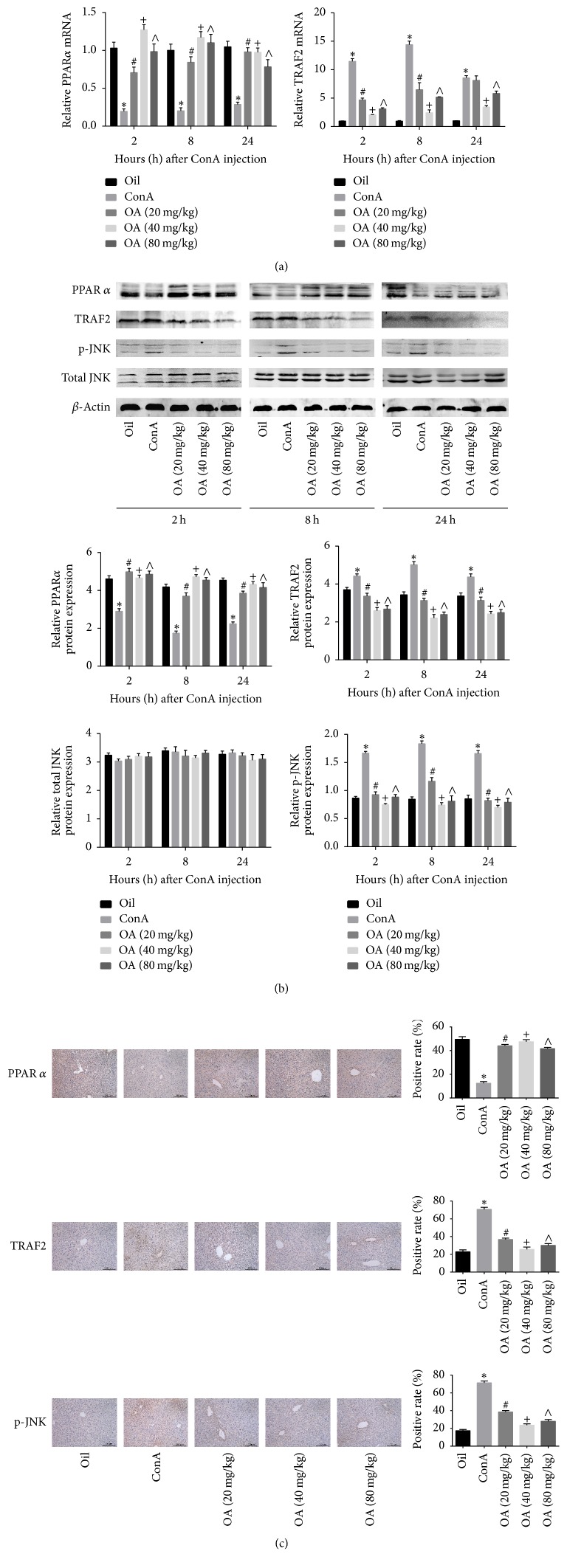
*OA regulates PPARα and JNK signaling in ConA-induced liver injury*.* Notes*. (a) mRNA expression levels of PPAR*α* and TRAF2 were measured with real-time PCR. (b) Protein expression levels of PPAR*α*, TRAF2, total JNK, and phospho-JNK were determined using western blot. (c) Representative immunohistochemistry images (200x magnification) were used to evaluate the expression levels of PPAR*α*, TRAF2, and phospho-JNK after 8 h of exposure to ConA (*n* = 6; ^*∗*^*P* < 0.05 for oil versus ConA; ^#^*P* < 0.05 for ConA + OA (20 mg/kg) versus ConA; ^+^*P* < 0.05 for ConA + OA (40 mg/kg) versus ConA; ^∧^*P* < 0.05 for ConA + OA (80 mg/kg) versus ConA).

**Figure 6:**
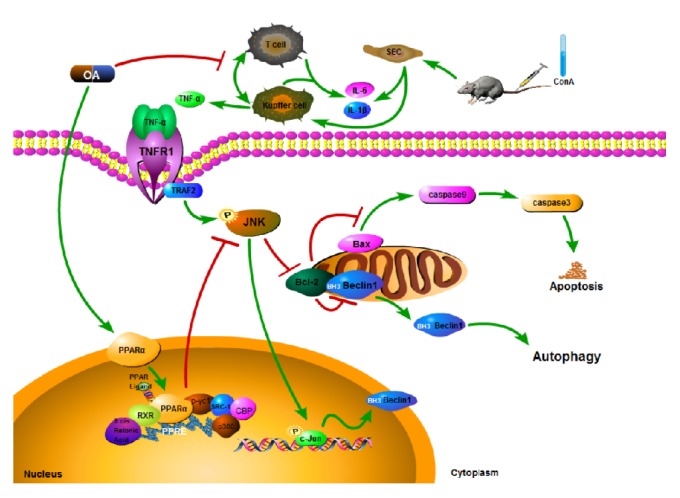
*A schematic representation of the pathways modulated by OA in response to ConA*. In our ConA-induced liver injury model, OA activates PPAR*α* leading to decreased expression of cytokines, including TNF-*α*, IL-1*β*, and IL-6. OA also attenuates JNK signaling activated by TNF-*α*. Furthermore, OA suppresses the phosphorylation of JNK. Activated JNK inhibits Bcl-2 activity, thus promoting the activation of caspase-9 induced by Bax expression and the activation of Beclin 1. OA exhibits hepatoprotective effects via attenuating apoptosis and autophagy through regulating JNK signaling and activating PPAR*α*.

We found that PPAR*α* protein and mRNA levels were significantly reduced in ConA treated mice, and pretreatment with OA abrogated this effect. The protein and mRNA levels of TRAF2 and the phosphorylation of JNK were markedly increased in the ConA group, and OA reduced the expression levels of TRAF2 and the activation of JNK (Figures 5(a)–5(c)). Our findings support the premise that OA has the ability to attenuate ConA-induced liver injury and that activation of PPAR*α* and suppression of JNK signaling may be one of the underlying mechanisms (Figure 6).

## 4. Discussion

OA is a pentacyclic triterpenoid found in a variety of medicinal herbs [14]. Due to its hepatoprotective properties, OA has been developed in China as a an oral remedy for the treatment of acute and chronic liver disorders [15]. Liu et al. [38] have shown that the hepatoprotective effects of OA are not evident until 24 h after exposure but are retained for at least 72 h. However, the underlying mechanisms of OA hepatoprotection during liver injury remain unknown.

Numerous animal studies have illustrated that OA protects against liver injury induced by small molecules like phalloidin, CCl4, and acetaminophen, by reducing serum transaminase levels and preventing necrosis [33, 34, 38]. To further investigate the pathways modulated by OA, we established a model of ConA-induced liver injury and verified the hepatoprotective effects of OA. In our study, ConA elevated serum aminotransferase levels, suggesting that ConA could damage hepatocytes and induce pathological lesions in mouse livers. Expectedly, OA attenuated the effects of ConA and alleviated ConA mediated liver injury and inflammation.

Wang et al. [8] have previously studied the mechanisms of ConA-triggered immune responses in mice. ConA was shown to bind the mannose gland on the surface of SECs, facilitating its binding to KCs. CD4^+^ Th cells can then identify the ConA-modified major histocompatibility complex presented by KCs and become activated, mediating the release of cytokines like TNF-*α*. SECs and KCs also secrete IL-1 and IL-6, which suggests that both KCs and SECs mediate liver injury along with CD4^+^ Th cells. Our study demonstrates that pretreatment with OA mitigates the inflammatory response mediated by ConA, as it reduced the levels of proinflammatory cytokines.

It has been shown that OA exerts its protective effects by binding and activating the PPAR nuclear receptor, PPAR*α* [39], which participates in the regulation of inflammation and immunity. Genetic ablation of PPAR*α* can promote NF-*Κ*B and c-jun activation in T lymphocytes, leading to increased production of IFN-*γ* and TNF, and lower expression levels of 2 Th2 cytokines [32]. We therefore hypothesized that OA can promote the expression of PPAR*α*. We found that ConA could, in fact, reduce both PPAR*α* mRNA and protein expression levels, an affect that was abrogated by pretreatment with OA.

TNF-*α* and IL-1*β* are known activators of JNK signaling [40–42]. TNFR2 binds TRAF2 directly, inducing TRAF2 degradation and activating JNK, which modulates gene transcription [43]. OA has been shown to inhibit the phosphorylation of JNK, attenuating B cell dysfunction and mitochondrial apoptosis [33, 44]. To discern the contribution of JNK signaling to OA's ability to alleviate ConA-induced liver injury, we assessed the phosphorylation of JNK and the expression levels of Bcl-2, Bax, Beclin 1, LC3B, caspase-3, and caspase-9 in liver tissue. When phosphorylated, JNK translocates from the cytoplasm to the nucleus, mediating the phosphorylation and activation of transcription factor c-Jun and upregulating expression of proapoptotic Bcl2-associated gene Bax and downregulating the expression of Bcl-2, thereby promoting apoptosis through caspase-9 and caspase-3 [45]. We found that phosphorylation of JNK and the expression of Bax, caspase-3, and caspase-9 were elevated in response to ConA, while the expression of Bcl-2 was reduced, effects that was reversed by OA. We therefore infer that OA attenuates apoptosis via inhibition of JNK in hepatocytes. Furthermore, JNK has been shown to promote the transcription of Beclin 1 [46]. Beclin 1, the first identified mammalian autophagy protein [47], has been reported to interact with the antiapoptotic protein Bcl-2 as well as other Bcl family members via its BH3 (Bcl-2 homology 3) domain, leading to inhibition of Beclin 1 activity and autophagy [48–50]. As an autophagic effector protein, LC3 (microtubule-associated protein 1 light chain 3) levels can modulate autophagic flux and can be used as a marker of autophagic activation [51–53]. OA pretreatment abrogated the increased expression levels of Beclin 1 and LC3 mediated by ConA, suggesting that OA alleviates liver injury by also inhibiting autophagy.

In conclusion, OA exhibits hepatoprotective effects via activation of PPAR*α* and inhibition of apoptosis and autophagy through inhibition of JNK signaling.

## Figures and Tables

**Table 1:** Oligonucleotide primer sequences used for qRT-PCR.

Gene	Forward (5′-3′)	Reverse (3′-5′)
*β-Actin*	GGCTGTATTCCCCTCCATCG	CCAGTTGGTAACAATGCCATGT
*IL-1β*	CGATCGCGCAGGGGCTGGGCGG	AGGAACTGACGGTACTGATGGA
*IL-6*	CTGCAAGAGACTTCCATCCAG	AGTGGTATAGACAGGTCTGTTGG
*TNF-α*	CAGGCGGTGCCTATGTCTC	CGATCACCCCGAAGTTCAGTAG
*PPARα*	AACATCGAGTGTCGAATATGTGG	CCGAATAGTTCGCCGAAAGAA
*Caspase-3*	ATGGAGAACAACAAAACCTCAGT	TTGCTCCCATGTATGGTCTTTAC
*Caspase-9*	TCCTGGTACATCGAGACCTTG	AAGTCCCTTTCGCAGAAACAG
*Bcl-2*	GCTACCGTCGTCGTGACTTCGC	CCCCACCGAACTCAAAGAAGG
*Bax*	AGACAGGGGCCTTTTTGCTAC	AATTCGCCGGAGACACTCG
*Beclin 1*	ATGGAGGGGTCTAAGGCGTC	TGGGCTGTGGTAAGTAATGGA
*LC3*	GACCGCTGTAAGGAGGTGC	AGAAGCCGAAGGTTTCTTGGG
*TRAF2*	AGAGAGTAGTTCGGCCTTTCC	AGAGAGTAGTTCGGCCTTTCC

**Table 2:** Pathological score for liver injury of 8 h.

	0	1	2	3	4	Mean
Oil	6	0	0	0	0	0.00
ConA	0	0	0	4	2	3.33^*∗*^
OA (20 mg/kg)	0	3	2	1	0	1.67^#^
OA (40 mg/kg)	1	4	1	0	0	1.00^+^
OA (80 mg/kg)	0	5	1	0	0	1.17^∧^

*Notes*. *n* = 6; ^*∗*^*P* < 0.05 for oil versus ConA; ^#^*P* < 0.05 for ConA + OA (20 mg/kg) versus ConA; ^+^*P* < 0.05 for ConA + OA (40 mg/kg) versus ConA; ^∧^*P* < 0.05 for ConA + OA (80 mg/kg) versus ConA.

## Supplementary Materials

Supplementary Materials To support our viewpoint that oleanolic acid is purely acting as a PPAR*α* ligand in this case, real-time PCR and western blot were used to evaluate the expression level of CPT1A and ACOX1, which were both target genes of PPAR*α*. As is shown in Supplemental Figure, the mRNA and protein expression of CPT1A and ACOX1 were both reduced in ConA group, and these levels were significantly upregulated in response to OA pretreatment. Our findings further indicated that OA exhibited liver protection by acting as a PPAR*α* ligand. OA is related to CPT1A and ACOX1 in ConA-induced liver injury. Notes: (A) mRNA expression levels of CPT1A and ACOX1 were measured with real-time PCR; (B) protein expression levels of CPT1A and ACOX1 were determined using western blot (*n* = 6; ^*∗*^*P* < 0.05 for oil versus ConA; ^#^*P* < 0.05 for ConA + OA (20 mg/kg) versus ConA; ^+^*P* < 0.05 for ConA + OA (40 mg/kg) versus ConA; ^∧^*P* < 0.05 for ConA + OA (80 mg/kg) versus ConA).
