# Supplementary figures and images for: The Hepatoprotection by Oleanolic Acid Preconditioning: Focusing on PPARα Activation
Source: PPAR Res. 2018 Apr 2;2018:3180396. doi: 10.1155/2018/3180396 (PMC5901823; doi:10.1155/2018/3180396)

Supplemental figure


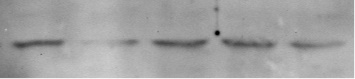

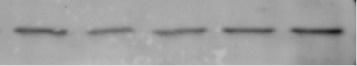

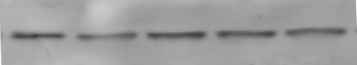

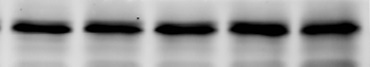

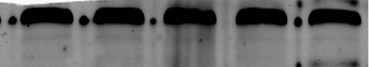

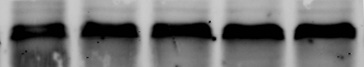

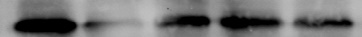

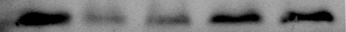

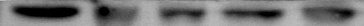










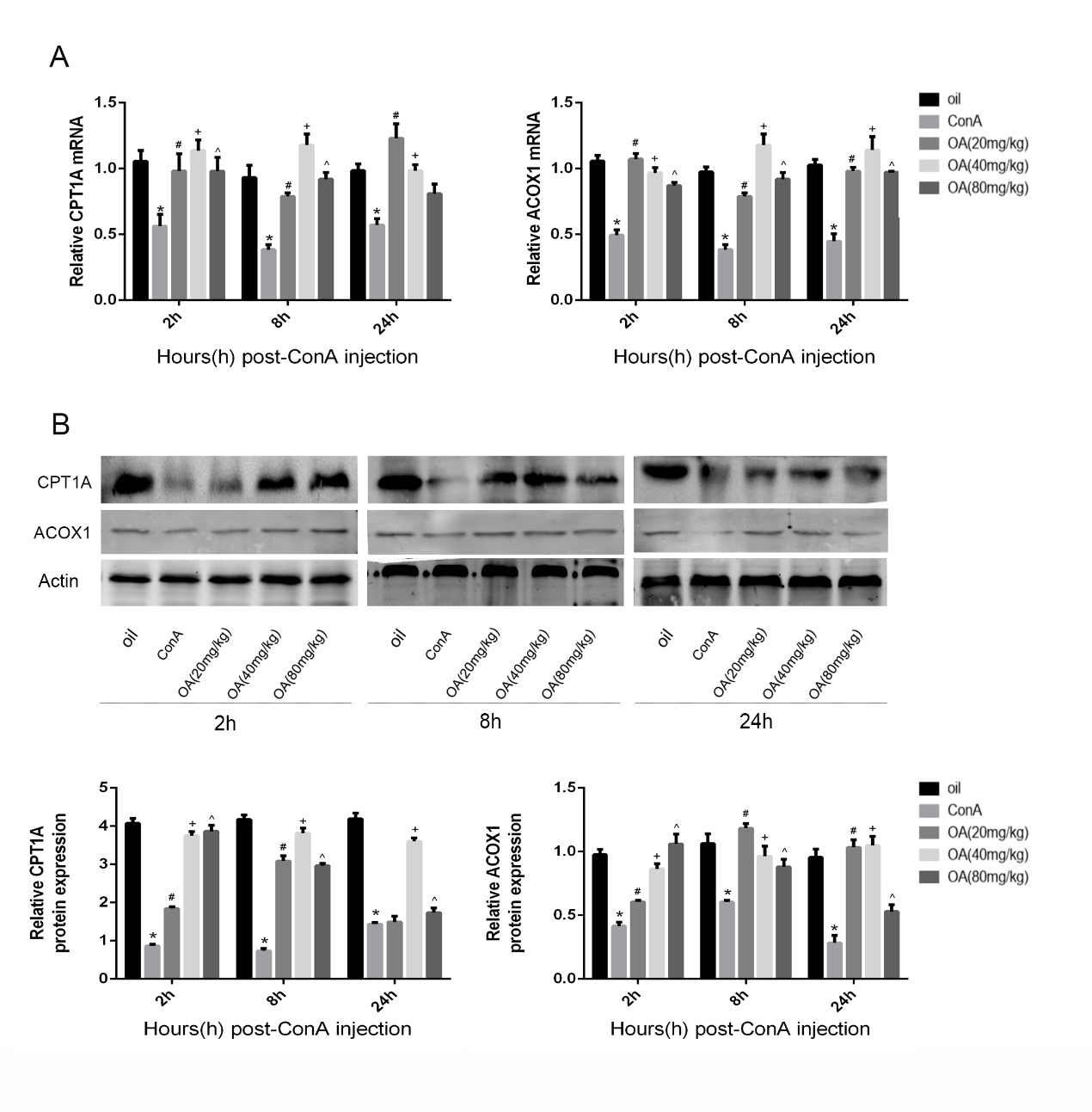

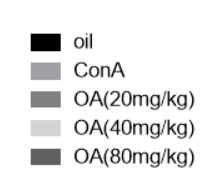

Supplement: Supplementary Materials — To support our viewpoint that oleanolic acid is purely acting as a PPARα ligand in this case, real-time PCR and western blot were used to evaluate the expression level of CPT1A and ACOX1, which were both target genes of PPARα. As is shown in Supplemental Figure, the mRNA and protein expression of CPT1A and ACOX1 were both reduced in ConA group, and these levels were significantly upregulated in response to OA pretreatment. Our findings further indicated that OA exhibited liver protection by acting as a PPARα ligand. OA is related to CPT1A and ACOX1 in ConA-induced liver injury. Notes: (A) mRNA expression levels of CPT1A and ACOX1 were measured with real-time PCR; (B) protein expression levels of CPT1A and ACOX1 were determined using western blot (n = 6; ∗P < 0.05 for oil versus ConA; #P < 0.05 for ConA + OA (20 mg/kg) versus ConA; +P < 0.05 for ConA + OA (40 mg/kg) versus ConA; ∧P < 0.05 for ConA + OA (80 mg/kg) versus ConA). [file 3180396.f1.docx]
